# Response to “Commentary on factors associated with diabetic foot ulcers and lower limb amputations in type 1 and type 2 diabetes supported by real‐world data from the German/Austrian DPV registry”

**DOI:** 10.1111/1753-0407.70036

**Published:** 2024-12-03

**Authors:** Alexander J. Eckert, Stefan Zimny, Marcus Altmeier, Ana Dugic, Anton Gillessen, Latife Bozkurt, Gabriele Götz, Wolfram Karges, Frank J. Wosch, Stephan Kress, Reinhard W. Holl, for the DPV‐Wiss‐Initiative

**Affiliations:** ^1^ Institute of Epidemiology and Medical Biometry University of Ulm Ulm Germany; ^2^ German Center for Diabetes Research (DZD) Munich‐Neuherberg Germany; ^3^ Department of General Internal Medicine, Endocrinology and Diabetology Helios Clinic Schwerin Schwerin Germany; ^4^ Klinik für Diabetologie, Klinikum Dortmund Dortmund Germany; ^5^ Medical Clinic I, Klinikum Bayreuth Friedrich‐Alexander‐University Erlangen‐Nürnberg Bayreuth Germany; ^6^ Department of Internal Medicine Herz‐Jesu‐Hospital Muenster Germany; ^7^ Department of Internal Medicine III and Karl Landsteiner Institute for Metabolic Disorders and Nephrology Clinic Hietzing, Vienna Health Care Group Vienna Austria; ^8^ Department of Internal Medicine, Diabetes, Gastroenterology, Tumor Medicine, and Palliative Care Academic Teaching Hospital Nürtingen Nurtingen Germany; ^9^ Clinic for Gastroenterology, Metabolic Disorders and Internal Intensive Medicine (Medical Clinic III), Department of Endocrinology and Diabetology University Hospital Aachen Aachen Germany; ^10^ Diabetespraxis Wosch Hanau Germany; ^11^ Diabetes, Sport and Physical Activity Working Group of the DDG Landau Germany; ^12^ Department of Internal Medicine I Vinzentius Hospital Landau Landau Germany

Dear Editor,

We thank the authors for this commentary and for considering our manuscript a valuable contribution to the topic of diabetic foot ulcers and lower limb amputations in adults with type 1 or type 2 diabetes.

Regarding the capturing of biomarkers and inflammatory markers, we want to point out that the listed parameters, namely, erythrocyte sedimentation rate (ESR), C‐reactive protein (CRP), neutrophil‐to‐lymphocyte ratio (NLR), platelet‐to‐lymphocyte ratio (PLR), the systemic inflammation response index (SIRI), and the systemic immune‐inflammation index (SII) are subject to a wide range of fluctuation. A blood sedimentation rate is nowadays rarely used in everyday practice, especially in outpatient care, as this marker is very unspecific. Of the above mentioned parameters, CRP alone is an acceptable predictor of systemic inflammation. The other indices or blood parameters from the differential blood count are not used clinically, neither in outpatient foot clinics nor in private practice, their relevance for treatment decisions is questionable. Further, not all of the included clinics and praxes in this study are specialized foot centres, and this is meaningful, since we want to provide data from everyday clinical practice in a real‐world setting. CRP might have been available in some, but not in all individuals included. The strength of our study on registry data is not to provide information on inconsistently raised parameters, but rather to provide well‐documented parameters and comorbidities that are available is most of the treated individuals as it is reflected in the real‐world care of these people. We agree, that some comorbidities might have been additionally investigated, but we wanted to focus on the most relevant and best captured information, in order to include as many individuals as possible in our analysis.

We agree, that there are some limitations in our study regarding wound classification. Again, we could only use what is routinely captured and documented in representative German‐speaking clinics and practices. Nevertheless, the Wagner wound classification is internationally accepted and widely used in Germany and in other countries.

Regarding the duration of DFU, we want to point out, that this consideration was the main reason, why we did not use regression models solely, but additionally did longitudinal Kaplan–Meyer analysis and calculated hazard ratios for amputations in order to include this time factor in the analysis. We also excluded individuals with amputations within a very short time (100 days) after the initial documentation of DFU for this specific analysis.

Lastly, we totally agree that Charcot foot is a risk factor for DFU, but the incidence of Charcot foot is below 1% in people with diabetes^1^ and would therefore be an independent topic for analysis.

In conclusion, we want to thank the authors for the critical and valuable response to our manuscript, and we will try to implement some of the mentioned parameters in future analyses on the topic of diabetic foot ulcers as far as practicable in our registry based setting.

## FUNDING INFORMATION

This study was supported through the German Federal Ministry for Education and Research within the German Centre for Diabetes Research (82DZD14E03). Further financial support was received by the German Robert Koch Institute and by the German Diabetes Association. Sponsors were not involved in data acquisition or analysis.
